# DRMDA: deep representations‐based miRNA–disease association prediction

**DOI:** 10.1111/jcmm.13336

**Published:** 2017-08-31

**Authors:** Xing Chen, Yao Gong, De‐Hong Zhang, Zhu‐Hong You, Zheng‐Wei Li

**Affiliations:** ^1^ School of Information and Control Engineering China University of Mining and Technology Xuzhou China; ^2^ School of Life Science Peking University Beijing China; ^3^ Xinjiang Technical Institute of Physics and Chemistry Chinese Academy of Science Ürümqi China; ^4^ School of Computer Science and Technology China University of Mining and Technology Xuzhou China

**Keywords:** miRNA, disease, miRNA–disease association, deep representation, auto‐encoder

## Abstract

Recently, microRNAs (miRNAs) are confirmed to be important molecules within many crucial biological processes and therefore related to various complex human diseases. However, previous methods of predicting miRNA–disease associations have their own deficiencies. Under this circumstance, we developed a prediction method called deep representations‐based miRNA–disease association (DRMDA) prediction. The original miRNA–disease association data were extracted from HDMM database. Meanwhile, stacked auto‐encoder, greedy layer‐wise unsupervised pre‐training algorithm and support vector machine were implemented to predict potential associations. We compared DRMDA with five previous classical prediction models (HGIMDA, RLSMDA, HDMP, WBSMDA and RWRMDA) in global leave‐one‐out cross‐validation (LOOCV), local LOOCV and fivefold cross‐validation, respectively. The AUCs achieved by DRMDA were 0.9177, 08339 and 0.9156 ± 0.0006 in the three tests above, respectively. In further case studies, we predicted the top 50 potential miRNAs for colon neoplasms, lymphoma and prostate neoplasms, and 88%, 90% and 86% of the predicted miRNA can be verified by experimental evidence, respectively. In conclusion, DRMDA is a promising prediction method which could identify potential and novel miRNA–disease associations.

## Introduction

MicroRNAs (miRNA) are a group of short non‐coding RNAs (20–25 nt) having important influence on post‐transcription level of gene expression. They bind to the 3′ untranslated regions (UTRs) and repress target mRNA translation 1, 2, 3. However, they can also up‐regulate gene expression in some situations. Recently, more and more evidence attach miRNAs with various human diseases 4. For instance, mir‐340 inhibited breast cancer cell migration and invasion through targeting oncoprotein c‐Met 5. Also, by targeting Cdc42 and Cdk6, miR‐137 inhibited the proliferation of lung cancer cells 6. What is more, miR‐211 promoted the progression of head and neck carcinomas by targeting TGFbeta R2 7. Therefore, predicting disease‐related miRNAs can promote biomarker identification, disease treatment and prevention 8. Also, the number of discovered miRNA accumulated quickly during the past 20 years 9, 10, 11. From the above, we can come to the conclusion that miRNA–disease association prediction becomes important and requires the help of computational methods 12.

Inspired by computational methods associating biomolecules with diseases 13, 14, 15, lots of computational models were established to predict miRNA–disease association, based on the assumption that miRNAs with similar functions are more likely to be associated with diseases with similar phonotypes 16, 17. Jiang *et al*. 18 built a hypergeometric distribution‐based model on the basis of disease phenotype similarity network, miRNA functional similarity network and known human disease–miRNA association network to identify unknown miRNA–disease associations. However, the model mostly relied on neighbour miRNA data, which greatly reduced its prediction accuracy. Later, Shi *et al*. 19 proposed a computational model using random walk algorithm on protein–protein interaction (PPI) network to predict new miRNA–disease associations. This model was based on the idea that one miRNA was more likely to be associated with a certain disease when it targeted genes which were related to that disease. In this way, they integrated PPI network, gene–disease associations and miRNA‐target interactions together to predict novel miRNA–disease associations. Mork *et al*. 20 took protein into consideration and presented miRPD method. In this method, with the help of protein–disease interactions and protein–miRNA interactions, both potential miRNAs and proteins associated with diseases can be predicted. Xu *et al*. 21 introduced a miRNA prioritization approach which could perform without known miRNA–disease associations. Instead of using known associations, they estimated the similarity between the targets of miRNAs and disease genes to identify potential associations. Nevertheless, all the models listed in this paragraph had the same limitation. They used miRNA–target interactions with high false‐positive and false‐negative samples, which could significantly reduce the prediction accuracy.

Based on the idea that similar miRNAs are more likely to be associated with similar diseases and vice versa, some other computational models without using miRNA–target interactions were proposed. Xuan *et al*. 22 introduced an HDMP model which calculated the miRNA–disease associations according to functional similarity of disease‐related miRNA's *k* most similar neighbours. Differ from previous studies, higher weight was assigned to miRNAs in the same cluster when calculating the miRNA functional similarity matrix as they are more likely to be associated with similar diseases. This similarity matrix was an integration of known miRNA–disease associations, disease phenotype similarity and disease semantic similarity based on disease term content. However, this property becomes a deficiency when applied to new diseases without any known related miRNAs and makes HDMP useless under this circumstance. Despite of that, HDMP is based on a local similarity measure rather than a global similarity measure which has higher efficiency. Chen *et al*. 23 presented a model based on global network similarity called RWRMDA, which predicted miRNA–disease associations according to integrated information of miRNA–miRNA functional similarity and known miRNA–disease associations. The transformation from local similarity measures to global similarity measures was the most important progression of RWRMDA. Although it performed better than former studies, RWRMDA fails to predict when facing new diseases with no related miRNAs. After adding Gaussian interaction profile kernel similarity into the algorithm, Chen *et al*. 24 proposed another model called WBSMDA. WBSMDA combined miRNA functional similarity, disease semantic similarity, miRNA–disease associations and Gaussian interaction profile kernel similarity for miRNAs and diseases to obtain potential disease–miRNA association. One shining point of WDSMDA is its capability of predicting related miRNAs for new diseases without known related miRNAs and related diseases for new miRNAs without known related diseases. To improve the previous algorithm, Chen *et al*. 25 introduced a model named HGIMDA. In this model, the miRNA functional similarity network of HGIMDA was a combination of miRNA functional similarity network and Gaussian interaction profile kernel similarities for miRNAs. Also, HGIMDA's disease similarity network was obtained in a similar way. In this way, the potential association between a disease and a miRNA could be inferred from an iterative equation which combined disease similarity network, miRNA functional similarity network and known miRNA–disease interaction. HGIMDA's good prediction performance had been verified.

Machine learning was used in several studies to predict novel miRNA–disease associations. For example, Xu *et al*. 26 built a miRNA–target‐dysregulated network (MTDN) which combined miRNA–target interactions and expression profiles of miRNAs and mRNAs. To deal with features extracted from information, support vector machine (SVM) classifier was implemented to separate positive miRNA–disease associations and negative ones in MTDN. However, the difficulty in obtaining negative miRNA–disease associations nowadays seriously decreases the accuracy when using the SVM classifier. Chen *et al*. 27 proposed a computational model called RLSMDA based on semi‐supervised learning, which calculated the semantic similarity between different diseases. RLSMDA is capable of predicting novel miRNA–disease associations and overcomes the problem of using negative associations between miRNAs and diseases. However, RLSMDA has difficulty in optimizing parameters and combining the classifiers from miRNA space and disease space together. Chen *et al*. 28 presented another method, RBMMMDA, based on restricted Boltzmann machine (RBM). RBM consists of layers of visible and hidden units and predicts miRNA–disease association types. When compared to previous methods, RBMMMDA's merit is that both new miRNA–disease associations and corresponding association types can be obtained. The trouble of RBMMMDA is that complex parameters are too difficult to learn.

In this study, we developed an efficient computational model called deep representations‐based miRNA–disease association (DRMDA) prediction. The motivation of this method was to find out the deep representation under the surface of disease semantic similarity, miRNA functional similarity and known miRNA–disease association. After deep representation, some noise within unprocessed data can be eliminated while features about association can be clearly presented. In this model, we built a stacked auto‐encoder composed of two visible layers and one hidden layer. Disease semantic similarity, miRNA functional similarity and known miRNA–disease similarity were integrated and deep represented in the stacked auto‐encoder. SVM was used as a classifier to sort out the true and false associations according to the outcome of auto‐encoder.

To evaluate the effectiveness of DRMDA, we introduced global leave‐one‐out cross‐validation (LOOCV), local LOOCV and fivefold cross‐validation. DRMDA achieved AUCs of 0.9177 and 0.8339 in global LOOCV and local LOOCV, respectively. And the average AUC of DRMDA in fivefold cross‐validation was 0.9156 ± 0.0006. Within the first group of case studies, 88% of top 50 predicted miRNAs for colon neoplasms, 90% of top 50 predicted miRNAs for lymphoma and 86% of top 50 predicted miRNAs for prostate neoplasms have been verified in recent experimental discoveries. Then in the second group of case studies, we transformed the miRNA–disease association matrix to make one certain disease a ‘new’ disease without known associated miRNAs. Under this condition, 96% of the top 50 predicted miRNAs for lung neoplasms were verified by recent experimental discoveries. The last group of case studies used an old edition of HDMM database, and the verification rate of breast neoplasms group was 84% in top 50 predicted miRNAs. All the results above have shown that DRMDA is an accurate way to infer new miRNA–disease associations, and has considerable advantage when compared with previous methods.

## Methods and materials

### Human miRNA–disease associations

The human miRNA–disease associations data used in DRMDA, which have been verified by experiments, were extracted from the latest version of HMDD database. The data set contains 383 human diseases, 495 miRNAs and 5430 miRNA–disease associations, which are transformed in to matrix *A* in the following way. If miRNA *m*(*j*) is associated with disease *d*(*i*), *A*(*i, j*) will be 1, otherwise 0. Furthermore, nd and nm represent the number of diseases and miRNAs in this study, which is 383 and 495, respectively.

### MiRNA functional similarity

From http://www.cuilab.cn/fles/images/cuilab/misim.zip, we downloaded miRNA functional similarity scores (calculated based on previous work 29) in January 2010, which are transformed into matrix FS, in which FS (*i, j*) stands for the functional similarity score between miRNA *m*(*i*) and *m*(*j*).

### Disease semantic similarity model 1

A disease can be described as a directed acyclic graph (DAG) which include *D*, the disease itself, *T*(*D*), both node *D* and its ancestor nodes, and *E*(*D*), the corresponding edges including the edges from parent nodes to child nodes directly. We calculate the semantic similarity value of disease *D* in model 1 as follows:

(1) $$D1\left(D\right)=\sum_{d\in T\left(D\right)}D_{D}\left(d\right)$$

(2) $$\left\{\begin{array}{cc} D_{D}\left(d\right)=1 & \text{if}\;d=D\\ D_{D}\left(d\right)=\max\{\Delta *D_{D}\left(d^{\prime }\right)|d^{\prime }\in \text{children of}\;d & \text{if}\;d\neq D \end{array}\right.$$

where Δ is the semantic contribution factor. If the distance between *D* and the other disease is shorter, the semantic contribution value will reduce less. As for *D* and *D*itself, there is no reduction and semantic contribution value is 1. And if disease terms have the same distance with *D*, they would have the same contribution to *D*1(*D*).

According to the presumption that two diseases are more similar if they share greater parts of their DAGs, we define disease *d*(*i*) and *d*(*j*)'s semantic similarly in model 1 as following function:

(3) $$\text{SS1}\left(d(i),d(j)\right)=\frac{\sum_{k\in T(d(i))\cap T(d(j))}\left(D_{d(i)}\left(k\right)+D_{d(j)}\left(k\right)\right)}{D1\left(d(i)\right)+D1\left(d(j)\right)}$$

in which SS1 stands for disease semantic similarity matrix based on the first computational model.

### Disease semantic similarity model 2

According to disease semantic similarity model 1 defined above, the disease terms having the same distance between disease *D* have the same contribution to the semantic value of disease *D*. However, different disease terms in the same layer of DAG may have different appearing frequency in DAGs of all diseases. For example, two diseases appear in the same layer of DAG of disease *D* and the first disease appears in less disease DAGs than the second disease. It is easy to conclude that the first disease is more specific than the second disease. Therefore, if the contribution of the first disease to the semantic value of disease *D* is assigned higher than the second, the algorithm will be more accurate according to the consideration above.

In conclusion, a more specific disease should have a greater contribution to the semantic value of disease *D*. So the contribution of disease term *d* to the semantic value of disease *D* in model 2 was defined as follows:

(4) $$D_{D}^{\prime }\left(d\right)=-\text{log}\left[\text{the number of DAGs including}\;d÷\text{the number of disease}\right]$$

Based on the presumption that two diseases are more similar if they share greater parts of their DAGs, we define disease *d*(*i*) and *d*(*j*)'s semantic similarly in model 2 as follows:

(5) $$\text{SS2}\left(d(i),d(j)\right)=\frac{\sum_{k\in T(d(i))\cap T(d(j))}\left(D_{d(i)}^{\prime }\left(k\right)+D_{d(j)}^{\prime }\left(k\right)\right)}{D2\left(d(i)\right)+D2\left(d(j)\right)}$$

where SS2 is the disease semantic similarity matrix based on the second computational model and *D2*(*d*(*i*)) and *D2*(*d*(*j*)) is the semantic value of disease *d*(*i*) and *d*(*j*), respectively. The entity SS2(*d*(*i*),*d*(*j*)) in row *i* column *j* is the disease semantic similarity between disease *d*(*i*) and *d*(*j*) based on disease semantic similarity model 2.

### Gaussian interaction profile kernel similarity for diseases

Gaussian interaction profile kernel similarity originates from the topological structure of the known miRNA–disease association network (inspired by literature 30). Based on the assumption that similar diseases are more likely to be associated with similar miRNAs, we define *IP*(*d*(*i*)) as the interaction profile of disease *d*(*i*) with each miRNA, that is the *i*th row of matrix *A*. The Gaussian interaction profile kernel similarities for diseases form matrix KD, and KD(*d*(*i*), *d*(*j*)) represents the similarity between disease *d*(*i*) and *d*(*j*). The following function calculates that similarity value:

(6) $$\text{KD}\left(d\left(i\right),d\left(j\right)\right)=\text{exp}\left(-\alpha_{d}||IP\left(d\left(i\right)\right)-IP\left(d\left(j\right)\right)|{|}^{2}\right)$$

where *α*_d_ is a parameter controlling the bandwidth of each kernel and originates from normalizing another bandwidth parameter $\alpha_{d}^{\prime }$ by the average number of associated miRNAs for all diseases. In this way, *α*_d_ was defined as the following function:

(7) $$\alpha_{d}=\frac{\alpha_{d}^{\prime }}{\frac{1}{\mathrm{nd}}\sum_{i=1}^{\mathrm{nd}}{||IP(d(i))||}^{2}}$$

### Gaussian interaction profile kernel similarity for miRNAs

The algorithm of Gaussian interaction profile kernel similarity for miRNAs is similar to that for diseases:

(8) $$\text{KM}\left(m(i),m(j)\right)=\exp\left(-\alpha_{m}||IP\left(m(i)\right)-IP\left(m(j)\right)|{|}^{2}\right)$$

(9) $$\alpha_{m}=\frac{\alpha_{m}^{\prime }}{\frac{1}{\mathrm{nm}}\sum_{i=1}^{\mathrm{nm}}{||IP(m(i))||}^{2}}$$

In this section, *IP*(*m*(*i*)) represents the whether miRNA *m*(*i*) is associated with each disease or not, that is the *i*th column of matrix *A*. Meanwhile, α_m_ is obtained by normalizing $\alpha_{m}^{\prime }$ by the average number of related diseases among all miRNAs.

### Integrated similarity for miRNAs and diseases

After the calculation above, the miRNA functional similarity, disease semantic similarity and Gaussian interaction profile kernel similarity are integrated to form the integrated similarity used in next step. Integrated similarity matrix SD for disease and matrix SM for miRNA are calculated as follows, respectively:

(10) $$\text{SD}\left(d(i),d(j)\right)=\left\{\begin{array}{cc} \text{SS}\left(d(i),d(j)\right) & d(i)\;\text{and}\;d(j)\;\text{has semantic}\\ \text{KD}\left(d(i),\;d(j)\right) & \text{otherwise}\;\;\;\;\text{similarity} \end{array}\right.$$

(11) $$\text{SM}\left(m(i),m(j)\right)=\left\{\begin{array}{cc} \text{FS}\left(m(i),m(j)\right) & m(i)\;\text{and}\;m(j)\;\text{has semantic}\\ \text{KM}\left(m(i),m(j)\right) & \text{otherwise}\;\;\;\;\text{similarity}\; \end{array}\right.$$

It should be noticed that the SS matrix here takes the average value of two kinds of disease semantic similarity matrix.

### DRMDA

**Figure 1:**
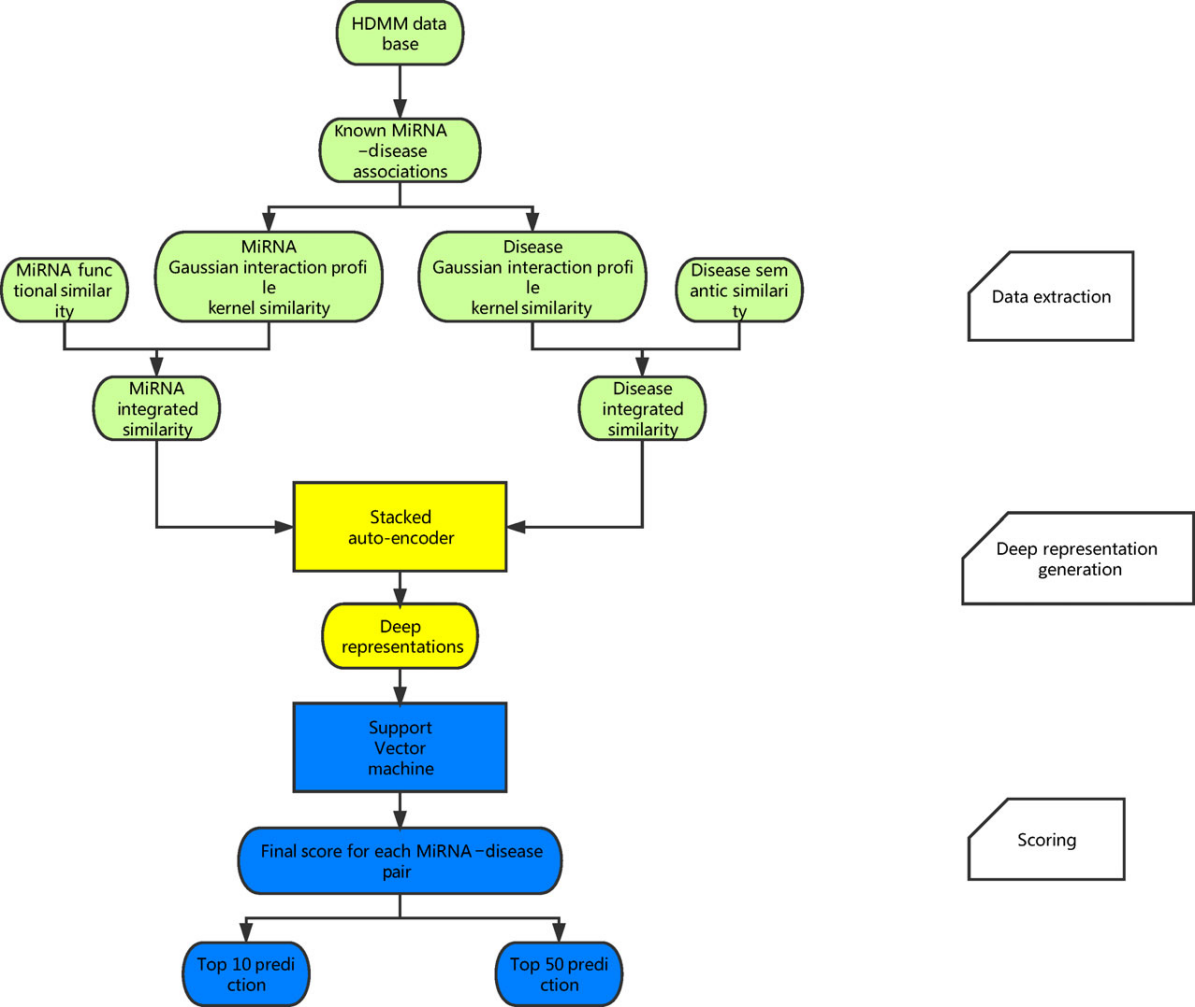
Flow chart of DRMDA model to obtain potential miRNA–disease associations according to the known associations in HMDD database.

The method named deep representations‐based miRNA–disease association (DRMDA) prediction was developed based on the assumption that similar diseases are associated with functionally similar miRNAs, which was similar to the basic assumptions used in the prediction for the interactions between drugs and target proteins 31, 32. DRMDA consists of three main steps (see Fig. 1): extracting data, generating deep representation and giving score by support vector machine (SVM).

In the first step, miRNAs' and diseases' information is obtained from disease semantic similarity, miRNA functional similarity and Gaussian interaction profile kernel similarity for disease and miRNA. As mentioned in the integrated similarity section, we combine these three similarities together and calculate integrated similarity matrix SD and matrix SM, which represents integrated disease and miRNA similarity, respectively. These two matrixes are used in the second step.

**Figure 2:**
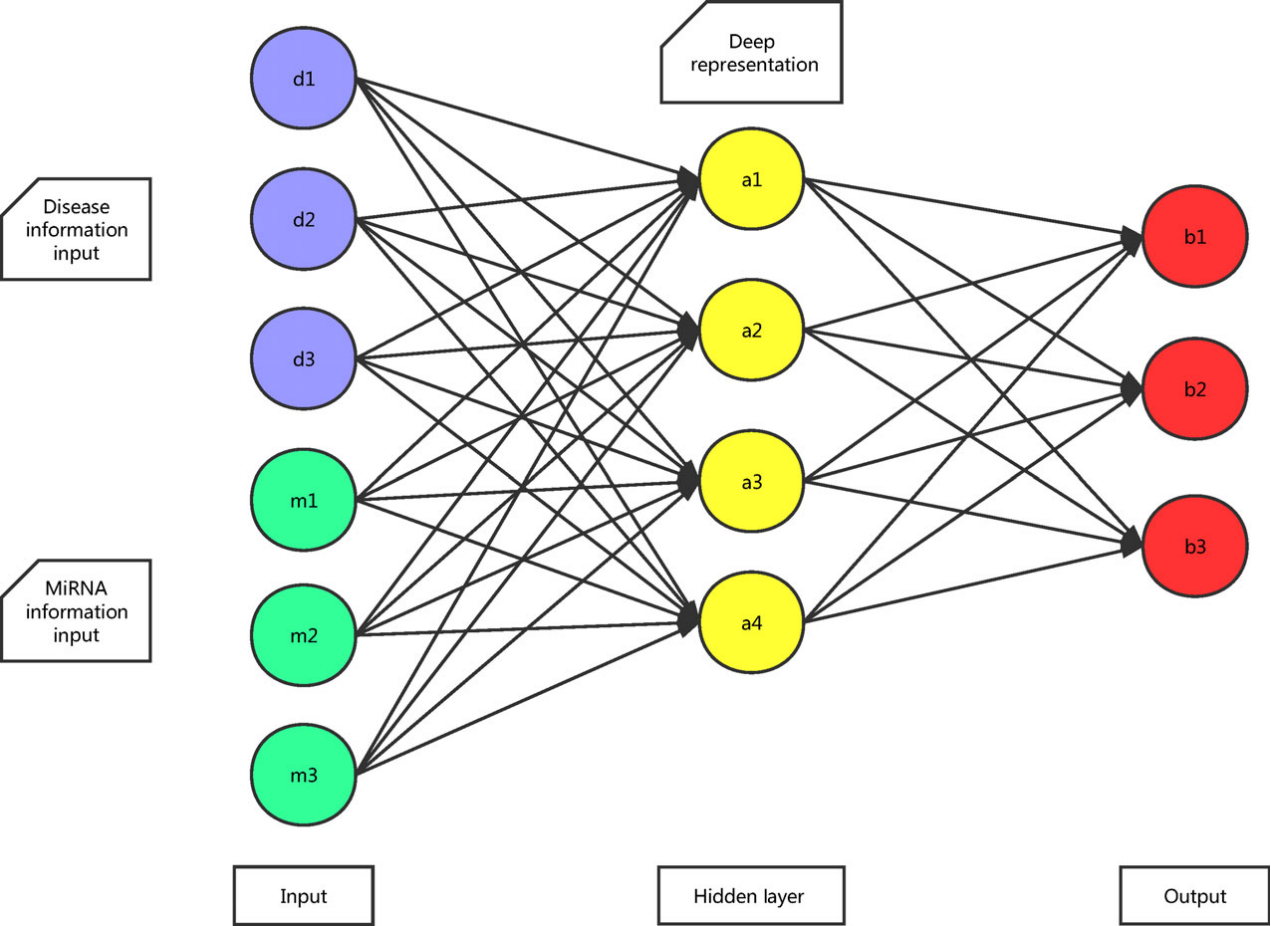
A stacked auto‐encoder composed of two visible layers and one hidden layer.

In the second step, all miRNA–disease associations can be represented by matrix T. If the *x*th known miRNA–disease association associates disease *d*(*i*) and miRNA *m*(*j*), the *x*th column of matrix T will consist of the *i*th column of matrix SD for disease *d*(*i*) and the *j*th column of matrix SM for miRNA *m*(*j*). So the number of columns in matrix T is the same as the number of positive associations, and the number of rows equals to the sum of the number of miRNAs and the number of diseases. Based on the literature 33, multilayer architecture neural network was built and trained with greedy layer‐wise unsupervised pre‐training algorithm 34. In this way, the dimension of matrix T is reduced after being processed. Meanwhile, valuable information is maximally preserved for next process and noise is filtered. As miRNA data and disease data have sparse distribution, sparse auto‐encoders were stacked in our neural network model (see Fig. 2, motivated by literature 35, 36).

A neural network consists of many computational units called ‘neuron’, each stands for an input vector *X =* (*x*_1_, *x*_2_,…, *x*_*n*_) and the output $k_{W,b}\left(x\right)=f\left(W^{T}x\right)=f\left(\sum_{i=1}^{n}W_{i}x_{i}+b\right)$. So the matrix *W* connects different neurons between neighbour layers. The sigmoid function is commonly used as an activation function between neighbour layers. Meanwhile, a conventional auto‐encoder would try to learn a function *k*_*W*,*b*_(*x*) ≈ *x*, which means it finds an approximation of the identity function to give an approximate output. The identity function seems a typically trivial function trying to learn but by placing constraints on the network. If the number of units in the first visible layer is *n*, which is the sum of number of diseases and number of miRNAs, and the number of units in the first hidden layer is set as *m*, after transformed through matrix *W* between these two layers, the *n*‐dimensional input will become a *m*‐dimensional vector which is a deep representation of the former one. Because of the special structure within the input vector, the reconstruct function can find the relationship among unprocessed information. The cost function of non‐sparse auto‐encoder is calculated as follows:

(12) $$J\left(W,b\right)=\left[\frac{1}{n}\sum_{i=1}^{n}\left(\frac{1}{2}||k_{W,b}\left(x^{\left(i\right)}\right)-\left(x^{\left(i\right)}\right)|{|}^{2}\right)\right]+\frac{\delta }{2}\sum_{h=1}^{n_{h}}\sum_{j=1}^{n_{j}}{\left(W_{hj}\right)}^{2}$$

where *J* (*W*,* b*) stands for the cost function, *x* ^(*i*)^ stands for the ith unit of the first layer, *n*_h_ and *n*_j_ stand for the number of rows and column of matrix *W* between the first and second layers, respectively. The first term makes *k*_*W*,*b*_(*x*) ≈ *x* and the second term prevents over‐fitting when *δ* balances the importance of these two terms. Normally, auto‐encoder is aimed to minimize *J*(*W*,*b*) so that output *k*_*W*,*b*_ (*x*) can approximate the raw data *x* as much as possible. Furthermore, large hidden units still could be used to discover valuable information if a new sparsity term was added to the overall cost function to complete sparse auto‐encoder as follows:

(13) $$J_{\mathrm{sparse}}\left(W,b\right)=J\left(W,b\right)+\beta \sum_{j=1}^{n_{2}}\text{KL}(\rho ||{\hat{\rho }}_{j})$$

(14) $${\hat{\rho }}_{j}=\frac{1}{n}\sum_{i=1}^{n}\left[a_{j}^{\left(2\right)}\left(x^{\left(i\right)}\right)\right]$$

(15) $$\text{KL}\left(\rho ||{\hat{\rho }}_{j}\right)=\rho \log\frac{\rho }{{\hat{\rho }}_{j}}+\left(1-\rho \right)log\frac{1-\rho }{1-{\hat{\rho }}_{j}}$$

where *β* alters the weight of sparsity penalty term, *ρ* is the sparsity parameter, *n*_2_ stands for the number of neurons in the second layer, a_*j*_^(2)^ stands for the *j*th neuron of the second layer and Eq. (13) is the Kullback–Leibler divergence between two Bernoulli random variables with different means.

The auto‐encoders were stacked layer by layer to form a network, which means the output of one layer is the input of the next layer. We constructed a three‐layer network and its parameters are shown in Table 1. Layer by layer, the original data gets its deep representation, and the network enriches useful information from original data.

**Table 1:** Parameters within stacked auto‐encoder

Parameters	Value
Neurons in layer 2	250
Neurons in layer 3	80
Weight of sparsity penalty term	5
Sparsity	0.05

Calculating association scores is the last step of DRMDA. SVM is a powerful classification algorithm originally developed by Vapnik *et al*. and it has been proved extremely effective in chemical and biological classifications 36, 37, 38. Firstly, negative miRNA–disease associations were randomly selected from miRNA–disease samples except positive miRNA–disease associations, and the number of negative associations is the same as positive associations. Then, the positive and the negative associations form matrix PT and NT in the same way as matrix T. Matrix PT and NT are processed by auto‐encoder whose parameters are learnt in the second step, and the results from auto‐encoder are used to train the classifier which originates from an open source package called LIBSVM 39. In this way, SVM is trained on all positive associations together with negative associations, which has the same number as positive associations. After training of SVM, a hyperplane for separation is calculated for the next step. Finally, miRNA–disease samples except positive miRNA–disease associations, also named as candidate samples, are scored. For each miRNA–disease candidate sample, the distance between the hyperplane for separation and the input data point is calculated. This distance determines the absolute value of the score of this miRNA–disease sample and which side of hyperplane the point is on decides whether the score is positive or negative. If a sample point is on the same side with most positive associations and has relative long distance with hyperplane, this sample will get a rather high score.

The parameters of SVM training and auto‐encoder are stored for later prediction. For prediction, all miRNA–disease samples except positive associations form a matrix *AT* in the same way as matrix T in the second step. Matrix *AT* is processed by auto‐encoder and SVM classifier, whose parameters have been learnt previously. Each sample gets a score after being processed, and for each disease, miRNAs are ranked by score. The higher a miRNA ranks in the list for a certain disease, the more likely that miRNA is associated with that disease. Within this step, the score of a candidate sample is compared with all other candidate samples, which includes those samples randomly selected as negative associations. According to previous study 36, the Radial Basis Functional (RBF) kernel had better performance than other kernels. However, the distance calculation is rather complicated when using RBF kernel. Our computational capacity cannot afford that, so the lineal kernel was used as substitute.

## Results

### Performance evaluation

Based on the known miRNA–disease associations in HMDD database, three validation schemas were used to evaluate the performance of DRMDA: global LOOCV, local LOOCV and fivefold cross‐validation. To compare DRMDA's performance with previous models, we selected five classical computational methods: HGIMDA 25, RLSMDA 27, HDMP 22, WBSMDA 24, RWRMDA 23 to compete with DRMDA in cross‐validation. Each known miRNA–disease association was regarded as test sample in turn while other known associations were treated as training samples. All of unknown miRNA–disease association were regarded as candidate samples. After processed by DRMDA model, each miRNA–disease pair would get a score. The score of the test sample was compared with the scores of all candidate samples in global LOOCV; however, test sample was only compared with candidates which contained the same disease in local LOOCV. In fivefold cross‐validation, the known miRNA–disease association list was randomly divided into five separate parts. One of the five parts would be selected as test samples in turn, while other parts were considered as training samples. The score of each miRNA–disease pair in the test part was compared with the scores of all candidate samples, respectively. This process was repeated for five times, so each association in the known miRNA–disease association list was compared with candidate samples once. In these three evaluation methods, whether the rank of test sample within candidate samples exceeded the preset threshold or not was the criterion of correctly prediction.

**Figure 3:**
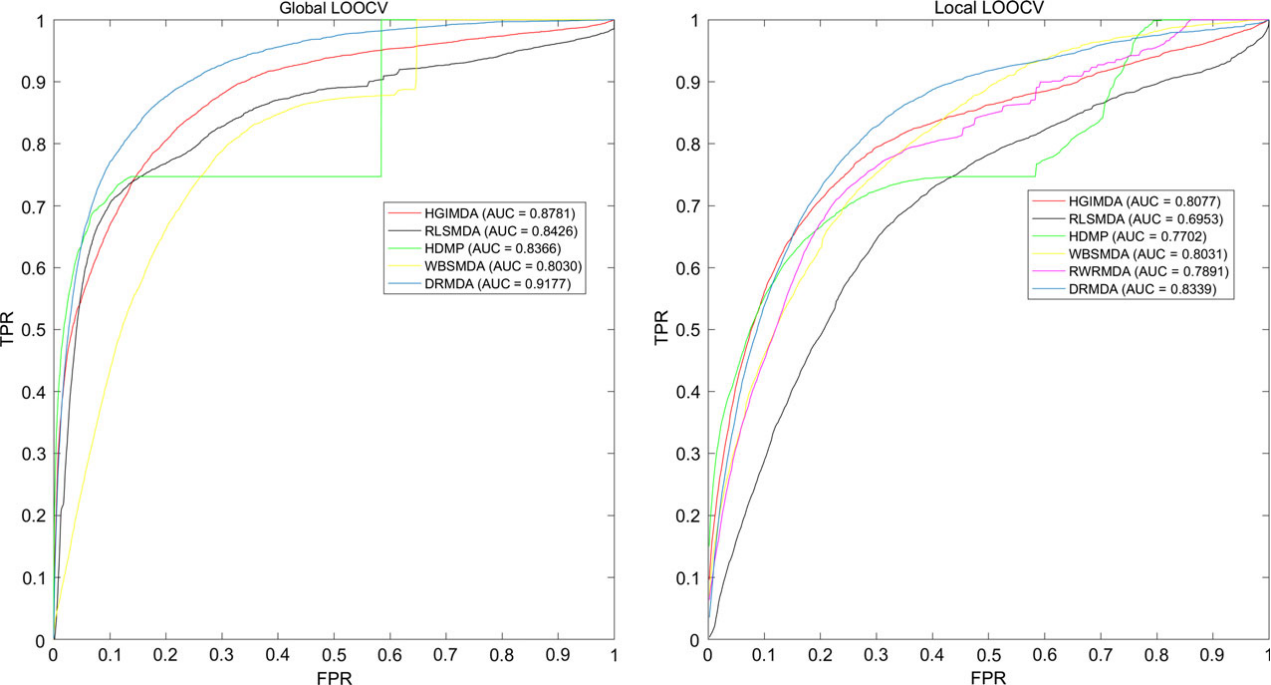
Performance comparison between DRMDA and five previous computational models (HGIMDA, RLSMDA, HDMP, WBSMDA and RWRMDA) in terms of ROC curve and AUC based on global and local LOOCV based on known miRNA–disease associations in the HMDD database. DRMDA's performance is significantly better than all the previous models to some extent and achieved AUC of 0.9177 in global LOOCV and 0.8339 in local LOOCV. Therefore, DRMDA proves to be efficient in predicting the potential miRNA–disease associations.

According to the data we calculated above, the receiver operating characteristic curve (ROC) was drawn to compare DRMDA and other five methods. The *x*‐axis stands for false‐positive rate (FPR, 1‐specificity), and specificity represents the rate of negative miRNA–disease associations whose ranks were lower than the threshold. The *y*‐axis stands for true‐positive rate (TPR, sensitivity), while sensitivity represents the percentage of positive miRNA–disease associations whose ranks exceeded the preset threshold. The area under the ROC curve (AUC) is a parameter to estimate the accuracy of the model. If AUC = 1, it means this model gets exactness rate of 100%, while AUC = 0.5 tell us that this model is predicting randomly. As a result, the AUC value in global LOOCV test of DRMDA, HGIMDA, RLSMDA, HDMP and WDSMDA was 0.9177, 0.8781, 0.8426, 0.8366 and 0.8030, respectively. As for local LOOCV, the AUC value of DRMDA, HGIMDA, RLSMDA, HDMP, WBSMDA and RWRMDA was 0.8339, 0.8077, 0.6953, 0.7702, 0.8031 and 0.7891, respectively (See Fig. 3). DRMDA, RLSMDA, HDMP and WBSMDA received an average AUC value of 0.9156 ± 0.0006, 0.8569 ± 0.0020, 0.8342 ± 0.0010 and 0.8185 ± 0.0009, respectively in fivefold cross‐validation. In conclusion, DRMDA is a more effective miRNA–disease association prediction method than previous methods.

### Case studies

To evaluate the prediction efficiency of DRMDA in real cases, three groups of case studies were implemented. In the first group of case studies, miRNA–disease associations originated from latest HDMM database were used as training samples and DRMDA would give score to every miRNA–disease sample based on training results. Then for each disease, miRNAs were ranked according to the score. In the second group, we altered the miRNA–disease matrix to make a certain disease a ‘new’ one in turn. So in this group of case studies, DRMDA must find out potential miRNAs related to this ‘new’ disease. The scores of all miRNA–disease samples containing this disease were calculated and ranked. Within the third group, another set of data including disease semantic similarity, miRNA functional similarity and known miRNA–disease similarity based on an old edition of HDMM database was used. But other steps in group three were the same as group one.

Colon neoplasms, also known as bowel cancer, are cancers developed from colon or the boundary of rectum 40. Effective ways to check it out in early stages are sigmoidoscopy or colonoscopy which is seldom done by patients and therefore hard to discover 41. Colon cancer is now the third most common cancer on Earth which accounts for 10% of the cases and one‐third of the people with this disease in the developed world died from it 42. So it is necessary to predict miRNAs associated with colon neoplasms. With the improvement of medical technology, more and more miRNAs related to colon neoplasms like hsa‐mir‐145 which targeted the insulin receptor substrate‐1 and repressed the growth of colon cancer cells were found. In our prediction for colon neoplasms, nine of top 10 miRNA predictions and 44 of top 50 miRNA predictions were verified by dbDEMC and miR2Disease database (see Table 2).

**Table 2:** Prediction of the top 50 potential miRNAs associated with colon neoplasms based on known miRNA–disease associations in HMDD database

miRNA	Evidence	miRNA	Evidence
hsa‐mir‐1	dbDEMC; miR2Disease	hsa‐mir‐206	dbDEMC
hsa‐mir‐21	dbDEMC; miR2Disease	hsa‐mir‐142	Unconfirmed
hsa‐mir‐133a	dbDEMC; miR2Disease	hsa‐mir‐203	dbDEMC; miR2Disease
hsa‐mir‐221	dbDEMC; miR2Disease	hsa‐let‐7a	dbDEMC; miR2Disease
hsa‐mir‐15a	dbDEMC	hsa‐let‐7i	dbDEMC
hsa‐mir‐146a	dbDEMC	hsa‐mir‐210	dbDEMC
hsa‐mir‐143	dbDEMC; miR2Disease	hsa‐mir‐19b	dbDEMC; miR2Disease
hsa‐mir‐222	dbDEMC	hsa‐mir‐223	dbDEMC; miR2Disease
hsa‐mir‐16	dbDEMC	hsa‐mir‐29a	dbDEMC; miR2Disease
hsa‐mir‐122	Unconfirmed	hsa‐mir‐27b	dbDEMC; miR2Disease
hsa‐mir‐15b	miR2Disease	hsa‐mir‐196a	dbDEMC; miR2Disease
hsa‐mir‐29c	dbDEMC	hsa‐let‐7b	dbDEMC; miR2Disease
hsa‐mir‐92a	Unconfirmed	hsa‐mir‐124	dbDEMC
hsa‐mir‐133b	dbDEMC; miR2Disease	hsa‐mir‐30a	miR2Disease
hsa‐mir‐155	dbDEMC; miR2Disease	hsa‐mir‐29b	dbDEMC; miR2Disease
hsa‐mir‐182	dbDEMC; miR2Disease	hsa‐let‐7g	dbDEMC; miR2Disease
hsa‐mir‐183	dbDEMC; miR2Disease	hsa‐let‐7e	dbDEMC
hsa‐mir‐150	Unconfirmed	hsa‐let‐7f	dbDEMC; miR2Disease
hsa‐mir‐181a	dbDEMC; miR2Disease	hsa‐mir‐199a	Unconfirmed
hsa‐mir‐18a	dbDEMC; miR2Disease	hsa‐let‐7c	dbDEMC
hsa‐mir‐20a	dbDEMC; miR2Disease	hsa‐let‐7d	dbDEMC
hsa‐mir‐125b	dbDEMC	hsa‐mir‐195	dbDEMC; miR2Disease
hsa‐mir‐19a	dbDEMC; miR2Disease	hsa‐mir‐181b	dbDEMC; miR2Disease
hsa‐mir‐146b	Unconfirmed	hsa‐mir‐34a	dbDEMC; miR2Disease
hsa‐mir‐31	dbDEMC; miR2Disease	hsa‐mir‐214	dbDEMC

Top 1–25 potential miRNAs are listed in the first column while top 26–50 potential miRNAs are listed in the second column.

A group of blood cell tumours originated from lymphocytes is called lymphoma. It consists of two main types: Hodgkin's lymphomas (HL) and the non‐Hodgkin lymphomas (NHL) 43. Recently, many miRNAs related to lymphoma have been found. For example, MiR‐200, which targeted cyclin E2, was commonly repressed in conjunctival MALT lymphoma 44. For the prediction for lymphoma, nine of top 10 miRNA predictions and 45 of top 50 miRNA predictions were verified by databases (see Table 3).

**Table 3:** Prediction of the top 50 potential miRNAs associated with lymphoma based on known miRNA–disease associations in HMDD database

miRNA	Evidence	miRNA	Evidence
hsa‐mir‐1	dbDEMC	hsa‐mir‐181b	dbDEMC
hsa‐mir‐221	dbDEMC	hsa‐let‐7i	dbDEMC
hsa‐mir‐133a	dbDEMC	hsa‐mir‐183	dbDEMC
hsa‐mir‐145	dbDEMC	hsa‐let‐7d	dbDEMC
hsa‐mir‐222	dbDEMC	hsa‐let‐7e	dbDEMC
hsa‐mir‐125b	Unconfirmed	hsa‐mir‐9	dbDEMC
hsa‐mir‐143	dbDEMC	hsa‐mir‐106b	dbDEMC
hsa‐mir‐34a	dbDEMC	hsa‐let‐7f	dbDEMC
hsa‐mir‐223	dbDEMC	hsa‐mir‐106a	dbDEMC
hsa‐mir‐29b	dbDEMC	hsa‐mir‐100	dbDEMC
hsa‐mir‐29a	dbDEMC	hsa‐let‐7g	dbDEMC
hsa‐mir‐199a	dbDEMC	hsa‐mir‐93	dbDEMC
hsa‐let‐7a	dbDEMC	hsa‐mir‐148a	dbDEMC
hsa‐mir‐146b	Unconfirmed	hsa‐mir‐192	dbDEMC
hsa‐mir‐30a	dbDEMC	hsa‐mir‐7	dbDEMC
hsa‐mir‐31	dbDEMC	hsa‐mir‐34b	dbDEMC
hsa‐mir‐182	dbDEMC	hsa‐mir‐25	dbDEMC
hsa‐let‐7b	dbDEMC	hsa‐mir‐205	dbDEMC
hsa‐mir‐142	Unconfirmed	hsa‐mir‐30b	dbDEMC
hsa‐mir‐214	dbDEMC	hsa‐mir‐141	dbDEMC
hsa‐let‐7c	dbDEMC	hsa‐mir‐30c	dbDEMC
hsa‐mir‐34c	Unconfirmed	hsa‐mir‐10b	dbDEMC
hsa‐mir‐196a	dbDEMC	hsa‐mir‐27a	dbDEMC
hsa‐mir‐195	dbDEMC	hsa‐mir‐375	Unconfirmed
hsa‐mir‐15b	dbDEMC	hsa‐mir‐206	dbDEMC

Top 1–25 potential miRNAs are listed in the first column while top 26–50 potential miRNAs are listed in the second column.

Prostate neoplasms, also known as carcinoma of the prostate, are cancers developed from the prostate. Prostate cancer is the second most common diagnosed cancer in men, but current diagnosis has low specificity 45. This indicates the importance of finding prostate neoplasm‐related miRNAs like miR‐145, whose target is proto‐oncogene ERG in prostate cancer. In the case study for prostate neoplasms, nine of top 10 miRNA predictions and 43 of top 50 miRNA predictions were verified by experimental evidence (see Table 4).

**Table 4:** Prediction of the top 50 potential miRNAs associated with prostate neoplasms based on known miRNA–disease associations in HMDD database

miRNA	Evidence	miRNA	Evidence
hsa‐mir‐1	dbDEMC	hsa‐mir‐19a	dbDEMC
hsa‐mir‐21	dbDEMC; miR2Disease	hsa‐mir‐214	dbDEMC; miR2Disease
hsa‐mir‐133a	dbDEMC	hsa‐mir‐196a	dbDEMC
hsa‐mir‐221	dbDEMC; miR2Disease	hsa‐mir‐29c	dbDEMC
hsa‐mir‐146a	miR2Disease	hsa‐mir‐199a	dbDEMC; miR2Disease
hsa‐mir‐15a	dbDEMC; miR2Disease	hsa‐mir‐223	dbDEMC; miR2Disease
hsa‐mir‐222	dbDEMC; miR2Disease	hsa‐mir‐17	miR2Disease
hsa‐mir‐122	Unconfirmed	hsa‐let‐7b	dbDEMC; miR2Disease
hsa‐mir‐15b	dbDEMC	hsa‐mir‐26b	dbDEMC; miR2Disease
hsa‐mir‐143	dbDEMC; miR2Disease	hsa‐mir‐210	miR2Disease
hsa‐mir‐16	dbDEMC; miR2Disease	hsa‐let‐7g	dbDEMC; miR2Disease
hsa‐mir‐133b	dbDEMC	hsa‐mir‐195	dbDEMC; miR2Disease
hsa‐mir‐150	dbDEMC	hsa‐mir‐206	dbDEMC
hsa‐mir‐92a	Unconfirmed	hsa‐mir‐30a	miR2Disease
hsa‐let‐7a	dbDEMC; miR2Disease	hsa‐mir‐203	Unconfirmed
hsa‐mir‐146b	Unconfirmed	hsa‐let‐7c	dbDEMC; miR2Disease
hsa‐mir‐155	dbDEMC	hsa‐mir‐30c	dbDEMC; miR2Disease
hsa‐mir‐182	dbDEMC; miR2Disease	hsa‐mir‐126	dbDEMC; miR2Disease
hsa‐let‐7e	dbDEMC	hsa‐mir‐19b	dbDEMC; miR2Disease
hsa‐let‐7f	dbDEMC; miR2Disease	hsa‐mir‐31	dbDEMC; miR2Disease
hsa‐let‐7i	dbDEMC	hsa‐mir‐142	Unconfirmed
hsa‐mir‐20a	miR2Disease	hsa‐mir‐181a	dbDEMC; miR2Disease
hsa‐mir‐18a	Unconfirmed	hsa‐mir‐181b	dbDEMC; miR2Disease
hsa‐let‐7d	dbDEMC; miR2Disease	hsa‐mir‐200b	Unconfirmed
hsa‐mir‐106a	dbDEMC; miR2Disease	hsa‐mir‐29b	dbDEMC; miR2Disease

Top 1–25 potential miRNAs are listed in the first column while top 26–50 potential miRNAs are listed in the second column.

The case studies above belong to the first group. Meanwhile, miRNA related to other diseases had been also predicted and ranked by score (see Table S1). The chart ranked all miRNA–disease samples by score, but the rank of miRNAs for a certain disease is more meaningful because the average scores are not the same for different diseases.

The second group was designed to validate the prediction accuracy of DRMDA, when dealing with new diseases without associated miRNAs. So all miRNA–disease associations of a certain disease were removed from miRNA–disease association matrix and the rest of associations were used for prediction. Here we used lung neoplasms as example, 48 of top 50 predicted miRNAs can be verified by at least one database among dbDEMC, HDMM and miR2Diseaes and all the top 10 predicted miRNAs can be verified (see Table 5). For instance, the miRNA having the biggest potential to be associated with lung neoplasms was hsa‐mir‐21. Experiments indicated that this miRNA repressed tumour suppressor PTEN and promoted growth and invasion in non‐small‐cell lung cancer 46.

**Table 5:** Prediction of the top 50 potential miRNAs associated with lung neoplasms based on known miRNA–disease associations in HMDD database within the second group of case study

miRNA	Evidence	miRNA	Evidence
hsa‐mir‐21	dbDEMC; HMDD; miR2Disease	hsa‐mir‐150	dbDEMC; HMDD; miR2Disease
hsa‐mir‐221	dbDEMC; HMDD; miR2Disease	hsa‐mir‐223	HMDD
hsa‐mir‐1	dbDEMC; HMDD; miR2Disease	hsa‐mir‐29b	dbDEMC; HMDD; miR2Disease
hsa‐mir‐146a	dbDEMC; HMDD; miR2Disease	hsa‐mir‐182	dbDEMC; HMDD; miR2Disease
hsa‐mir‐155	dbDEMC; HMDD; miR2Disease	hsa‐let‐7a	dbDEMC; HMDD; miR2Disease
hsa‐mir‐222	dbDEMC; HMDD	hsa‐mir‐181a	dbDEMC; HMDD
hsa‐mir‐125b	HMDD; miR2Disease	hsa‐mir‐206	HMDD
hsa‐mir‐20a	dbDEMC; HMDD; miR2Disease	hsa‐mir‐486	dbDEMC; HMDD
hsa‐mir‐15a	dbDEMC	hsa‐mir‐146b	HMDD; miR2Disease
hsa‐mir‐16	dbDEMC; miR2Disease	hsa‐mir‐15b	dbDEMC
hsa‐mir‐17	HMDD; miR2Disease	hsa‐mir‐181b	dbDEMC; HMDD
hsa‐mir‐92a	HMDD	hsa‐mir‐9	HMDD; miR2Disease
hsa‐mir‐18a	dbDEMC; HMDD; miR2Disease	hsa‐let‐7b	HMDD; miR2Disease
hsa‐mir‐133a	dbDEMC; HMDD	hsa‐let‐7i	dbDEMC; HMDD
hsa‐mir‐19a	dbDEMC; HMDD; miR2Disease	hsa‐mir‐26b	dbDEMC; HMDD
hsa‐mir‐143	dbDEMC; HMDD; miR2Disease	hsa‐mir‐199a	dbDEMC; HMDD; miR2Disease
hsa‐mir‐145	dbDEMC; HMDD; miR2Disease	hsa‐mir‐200b	dbDEMC; HMDD; miR2Disease
hsa‐mir‐19b	dbDEMC; HMDD	hsa‐mir‐328	dbDEMC
hsa‐mir‐29c	dbDEMC; HMDD; miR2Disease	hsa‐mir‐31	dbDEMC; HMDD; miR2Disease
hsa‐mir‐133b	dbDEMC; HMDD; miR2Disease	hsa‐mir‐203	dbDEMC; HMDD; miR2Disease
hsa‐mir‐126	dbDEMC; HMDD; miR2Disease	hsa‐mir‐24	HMDD; miR2Disease
hsa‐mir‐122	Unconfirmed	hsa‐let‐7e	HMDD; miR2Disease
hsa‐mir‐34a	dbDEMC; HMDD	hsa‐mir‐208a	Unconfirmed
hsa‐mir‐29a	dbDEMC; HMDD; miR2Disease	hsa‐mir‐483	dbDEMC
hsa‐mir‐142	HMDD	hsa‐mir‐26a	dbDEMC; HMDD; miR2Disease

Top 1–25 potential miRNAs are listed in the first column while top 26–50 potential miRNAs are listed in the second column.

To make sure DRMDA was effective when using other databases, an old edition of HDMM database was used in the third group. We altered the number of neurons in each layer to adapt the old database and predicted top 50 potential miRNAs for breast neoplasms. Nine of top 10 miRNA predictions and 42 of top 50 miRNA predictions were verified by at least one database among dbDEMC, HDMM and miR2Diseaes (see Table 6).

**Table 6:** Prediction of the top 50 potential miRNAs associated with prostate neoplasms based on known miRNA–disease associations in HMDD database

miRNA	Evidence	miRNA	Evidence
hsa‐mir‐130b	dbDEMC	hsa‐mir‐208b	Unconfirmed
hsa‐mir‐449b	Unconfirmed	hsa‐mir‐154	dbDEMC
hsa‐mir‐382	dbDEMC	hsa‐mir‐561	Unconfirmed
hsa‐mir‐500	dbDEMC	hsa‐mir‐99b	dbDEMC
hsa‐mir‐532	dbDEMC	hsa‐mir‐208	dbDEMC
hsa‐mir‐124	dbDEMC; HMDD	hsa‐mir‐92b	dbDEMC
hsa‐mir‐498	dbDEMC	hsa‐mir‐660	dbDEMC
hsa‐mir‐301a	HMDD	hsa‐mir‐501	dbDEMC
hsa‐mir‐431	dbDEMC	hsa‐mir‐377	dbDEMC
hsa‐mir‐224	dbDEMC; HMDD	hsa‐let‐7e	dbDEMC; HMDD
hsa‐mir‐363	dbDEMC	hsa‐mir‐494	Unconfirmed
hsa‐mir‐486	dbDEMC; HMDD	hsa‐mir‐659	dbDEMC
hsa‐mir‐139	dbDEMC; HMDD	hsa‐mir‐376b	dbDEMC
hsa‐mir‐370	dbDEMC	hsa‐mir‐16	dbDEMC; HMDD
hsa‐mir‐26b	dbDEMC; HMDD	hsa‐mir‐150	dbDEMC
hsa‐mir‐487b	dbDEMC	hsa‐mir‐136	dbDEMC; miR2Disease
hsa‐mir‐190	dbDEMC	hsa‐mir‐526b	dbDEMC
hsa‐mir‐297	Unconfirmed	hsa‐mir‐100	dbDEMC; HMDD
hsa‐mir‐22	dbDEMC; HMDD; miR2Disease	hsa‐mir‐512	Unconfirmed
hsa‐mir‐323	dbDEMC	hsa‐mir‐409	HMDD
hsa‐mir‐381	dbDEMC	hsa‐mir‐148b	dbDEMC; HMDD
hsa‐mir‐518b	Unconfirmed	hsa‐mir‐301b	HMDD
hsa‐mir‐33a	Unconfirmed	hsa‐mir‐615	dbDEMC
hsa‐let‐7c	dbDEMC; HMDD	hsa‐mir‐183	dbDEMC; HMDD
hsa‐mir‐337	dbDEMC	hsa‐mir‐365	dbDEMC; miR2Disease

Top 1–25 potential miRNAs are listed in the first column while top 26–50 potential miRNAs are listed in the second column.

## Discussion

Potential associations between miRNAs and diseases are being identified by researchers from the fields of bioinformatics or medical science. Compared with traditional methods, building a computational model dealing with heterogeneous biological big data is less expensive and more powerful. To fulfil the requirement of predicting potential miRNA–disease associations, we proposed a computational model called DRMDA. This algorithm calculated the score of each miRNA–disease sample by analysing known miRNA–disease interactions, disease semantic similarity and miRNA functional similarity. Then, potential associations were selected according to the score. Within the test of global LOOCV, local LOOCV and fivefold cross‐validation, DRMDA got pretty high score when compared to previous methods. Furthermore, when examined by experimental literatures in miR2Diseaes and dbDEMC databases, the verification rate of the top 50 miRNA predictions for colon neoplasms, lymphoma and prostate neoplasms in the first group of case studies reached 88%, 90% and 86%, respectively. And in the second group of case studies, 96% of the top 50 miRNA predictions for lung neoplasms were verified by experimental evidence in databases. Meanwhile, 84% of the top 50 miRNA predictions for breast neoplasms were verified in the third group of case studies. Both cross‐validation and case studies had proved the effectiveness of DRMDA in predicting potential miRNA–disease interactions.

The success of DRMDA can be concluded as follows. First of all, DRMDA is the first algorithm that uses a deep representation stacked auto‐encoder core to predict miRNA–disease associations. Lots of noise within disease semantic similarity matrix and miRNA functional similarity matrix are filtered by sparse auto‐encoder. High‐dimension vectors with much noise can be transformed by DRMDA into low‐dimension vectors which are easier for SVM to classify. Secondly, HMDD database provides plenty of known miRNA–disease associations for DRMDA and guarantees the effectiveness of the model. Finally, DRMDA adopts some good algorithms from previous methods. For example, the disease semantic similarity matrix in DRMDA is an average result of two kinds of algorithms. These improvements make DRMDA a better method than previous ones.

However, DRMDA still has its deficiencies. Firstly, DRMDA uses SVM after the deep representation step, which means negative miRNA–disease associations must be used in the model. Due to the difficulty in obtaining negative associations, this procedure reduces accuracy. Secondly, it is not easy to optimize the complex parameters in the DRMDA. Finally, the SVM kernel function used in this model is a linear one because of computing power limit. A radial basis function (RBF) kernel SVM classifier takes more time, but may perform better. Our method aimed to find out miRNA–disease associations and to predict cancer risk; however, as what has been pointed out in the literature 47, using a single disease‐related miRNA to judge cancer risks for all the persons may have imprecise results. So based on each person's miRNA profiles, we planned to construct various cancer hallmark networks to effectively evaluate cancer risks 47. In this way, three important problems in the personalized medicine could be considered within future studies 47, 48, which are obtaining the tumour recurrence and metastases probability, predicting potential consequences after applying a specific drug to the patients and identifying molecular signatures to evaluate and predict therapeutic results after cancer treatment in the framework of miRNAs.

## Conflict of interest

The authors declare no conflict of interests.

## Supporting information


**Table S1** We implemented DRMDA to calculate the score of all candidate miRNA–disease pairs when all the known miRNA–disease associations in HMDD database were regarded as training samples. This prediction result is published for further experimental validation and research.
